# The proximal promoter region of *mTert *is sufficient to regulate telomerase activity in ES cells and transgenic animals

**DOI:** 10.1186/1477-7827-4-5

**Published:** 2006-02-03

**Authors:** Eva Pericuesta, Miguel Angel Ramírez, Ana Villa-Diaz, Aroa Relaño-Gines, Juan Maria Torres, Marta Nieto, Belen Pintado, Alfonso Gutiérrez-Adán

**Affiliations:** 1 Departamento de Reproducción Animal y Conservación de Recursos Zoogenéticos, INIA, Ctra. De La Coruña Km 5,9, Madrid 28040, Spain; 2 Centro de Investigación en Sanidad Animal (CISA-INIA), Ctra. de Valdeolmos a El Casar, Valdeolmos, 28130, Madrid, Spain; 3 Departamento de Biología Molecular y Celular. Centro Nacional de Biotecnología. Consejo Superior de Investigaciones Científicas. E-28049 Madrid, Spain

## Abstract

**Background:**

The reverse transcriptase of telomerase (Tert) controls telomerase activity maintaining the end of linear chromosomes in eukaryotic cells. Telomerase function is highly active in undifferentiated multipotent stem cells, decreases with cell differentiation and is generally absent from most somatic cells in the adult. Its absence is responsible of telomeres shortening in such somatic cells. Using an in vivo transgenic model and an in vitro culture differentiation of adult stem cells, we examined the elements of the mouse Tert (mTert) promoter that control telomerase activity.

**Results:**

Three constructs comprising 1, 2 or 5 kb of the mTert promoter sequence coupled to the coding sequence of the green fluorescent protein (EGFP) were electroporated into embryonic stem (ES) cells. Transformed ES cells were able to mimic the expected mTert expression, which was associated to green fluorescence. One and 5 kb promoter produced the higher expression of EGFP, on ES cells. When ES cells were allowed to differentiate to embryoid bodies and to other cell types, they lost gradually the expression of mTert-EGFP as consequence of differentiation. No differences were found among the three constructs analyzed. We then generated transgenic mice with the three constructs. Expression of the reporter gene was monitored by reverse transcription-PCR analysis and EGFP visualization. The mRNA expression of the three constructs was lower than the endogenous mTert, but mimicked the endogenous mTert transcription pattern; however, no fluorescent expression of EGFP was detected in adult tissues. EGFP expression of the three constructs was visualized at the blastocysts stage and in new ES cells generated from them; in the germinal ring of E13 dpc foetuses; in ES-like colonies and in germinal stem cells generated from neonatal and adult testis cells; and in neuroesferes generated from E14 dpc foetuses' brain cells.

**Conclusion:**

The 1 kb promoter upstream of the initiating ATG codon of mTert contains all the regulatory elements to control telomerase expression in ES cells during in vitro loss of pluripotency. The transgenic mouse lines generated represent an appropriate system to analyze the expression of mouse Tert gene under physiological condition and during establishment of stem cell lines generated from embryonic or adult tissues.

## Background

**Figure 1:**
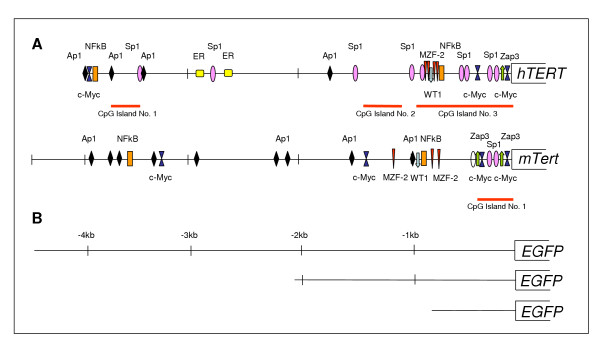
Promoter and regulators elements of the transcriptional of human and mouse *TERT*. CpG island in human and mouse *TERT *promoter identify using the CpG Island Explorer Program at  are indicated in red line **(A)**. Construct 5 k contain 4.5 Kb of the mouse promoter region, construct 1 k contains the proximal region of the promoter where all the transcription activator elements of the promoter reside (4 regions of c-Myc binding and two regions recognized by proteins from the sp1 family) and construct 2 k, includes, in addition to this proximal region, two of the three regions of MZF2 (myeloid zinc finger protein) binding, that reduces the transcriptional activity of the promoter **(B)**.

Embryonic stem (ES) cells are undifferentiated cells that, like somatic stem cells, possess the unique ability of self-renewal and multilineage differentiation. ES cells are derived from the inner cell mass of a developing pre-implantation embryo. Unlike most somatic cells, ES cells are spontaneously immortal and appear capable of indefinite self-renewal while retaining their ability to differentiate and to contribute to the germ line after blastocyst injection. The ability to self-renew indefinitely is lost as ES cells differentiate to generate somatic tissue. One mechanism that may underlie the capability of stem cells to replicate indefinitely is the expression of the DNA repair enzyme complex that includes telomerase, an RNA-dependent DNA polymerase that maintains telomere length [1]. Analysis of telomerase enzyme levels and activity has shown that high levels are expressed by ES cells [2] while lower levels are expressed in proliferative cells of renewal tissues. In addition, telomerase activity is down-regulated in cells that exit the cell cycle via either terminal differentiation or (reversible) quiescence. The key factor that controls the activity of the telomerase is the reverse transcriptase of telomerase (*Tert*) [2]. In contrast to most somatic cells, stem, germ and tumour cells have high telomerase activity through *Tert *transcriptional up-regulation. One way of regulating telomerase activity appears to be to regulate gene expression, and studies have begun to identify key regulators. The human and mouse *Tert *promoters were cloned in 1998 [3]. The core *Tert *promoter, a region of 300 bp upstream of the transcriptional start site, lacks a TATA sequence, but contains two E-boxes surrounding several Sp1 binding sites. E-box sites bind several cellular proteins, including the Myc/Mad/Max family of transcription factors [3] (Fig. 1).

**Figure 2:**
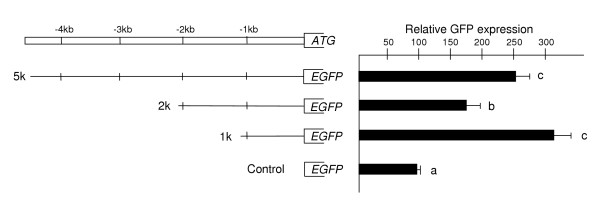
*mTert *promoter activity in R1 ES cells. On the left, the size of the *mTert*-pormoter-EGFP reporter construct is shown. Cells transformations with an EGFP plasmid without promoter are used as controls for each transfection. On the right, the relative GFP expression of the three promoter construct, and the standard deviation is indicated by solid bars. The GFP intensity produced by the promoterless construct was normalized to value of 100; GFP intensity of other constructs is shown relative to this control. Results are expressed as the mean of at least three independent experiments. A, b, c indicate p < 0.05 in a one-tailed unpaired X^2^-test.

There are evidences that telomerase plays important roles in the regulation of cell proliferation, differentiation, and survival. Examples include how over expression of *hTERT *can immortalize cultured fibroblasts and epithelial cells [4]; the down-regulation of telomerase during muscle cell differentiation [5]; and that *Tert *promotes cell survival (prevents apoptosis) of developing mouse and rat brain neurons [6]. High levels of *Tert *and telomerase activity have been shown in several types of stem cells including embryonic stem cells [7,8], hematopoietic stem cells [9], and neural progenitor cells [10]. A progressive decrease in telomerase levels appears to occur in association with successive lineage restriction and cellular differentiation, suggesting that telomerase could play an important role in controlling cell fate and both human and mouse ES cells maintain high telomerase activity [11,12]. Telomerase activity is down-regulated during differentiation of rodent and human ES cells [13,14] as well as in embryonal carcinoma (EC) cells [15]. This suppression of telomerase activity in differentiated EC cells requires histone deacetylation in early *hTERT *gene down-regulation and DNA methylation for maintenance of silencing of the *hTERT *gene [15]. Both histone deacetylases (HDACs) and methylation are tightly regulated in ES cells [16].

Current knowledge of *Tert *regulation and telomerase activity very largely derives from studies of neoplasic and/or immortalised cell lines. Although useful for understanding cancer models, such studies may be less relevant to normal stem cells and differentiation, and hence the need to study telomerase regulation in normal development. Recently it has been developed a transgenic mouse model using the human *TERT *promoter to express bacterial *LacZ *[17]. In two of the three transgenic lines generated *LacZ *expression was detectable only in testes, while in the other line the pattern of expression was different from the endogenous mouse *Tert*. We investigated the regulatory elements of the mice mTert promoter, using EGFP as a live reporter of expression, to address a detailed analysis of this promoter activity under physiological condition *in vivo *and under stem cell generation. The aims of this report were (i) to determine the region of the *mTert *promoter that regulates telomerase activity during differentiation of ES cells *in vitro *and in adult mice; (ii) to analyse the regulation of *mTert *during differentiation of ES cells; (iii) to study the regulation of the *mTert *promoter in an *in vivo *system; and (iv) to produce a reporter system that identifies newly formed stem cell lines from embryonic, foetal, newborn, and adult tissues.

## Materials and methods

### Bioinformatics analysis

Homology searches were carried out using BLAST (Basic Local Alignment Search Tool): . The search for putative transcription factor binding site was carried our using the Lasergene Sequence Analysis Software (DNASTART, Inc, Wisconsin, USA). The CpG island was searched using the CpG Island Explorer Program at [18].

### Generation of mTert-EGFP construct

Three constructs were generated using the available sequence of 4471 bp of the promoter of mouse *mTert *gene (NCBI Gene Banck; accession number AF 121949). Three fragments of the mTert promoter of 1086, 1846, and 4471 bp, downstream of the first ATG of the open reading frame of *mTert *gene, were amplified by PCR and ligated to pEGFP (Clontech Laboratories, Inc., Palo Alto, CA, USA) to promote the expression of EGFP. Orientation and sequence of each insert was checked by sequencing. The constructs were named 1 k-, 2 k-, and 5 k-*mTert*-EGFP to respectively indicate the construct with 1086, 1846 or 4471 bp of the *mTert *promoter. The plasmids were amplified and linearized before being used for electroporation of ES cell.

### Culture, transformation and differentiation of ES Cells

Undifferentiated mouse ES cells (R1 from A. Nagy lab. and MAR1 a B6DF1 origin established by our group) were maintained on mitomycin-C treated (Sigma-Aldrich corporation St. Louis, MO, USA) mouse embryonic fibroblast (MEF) cells in 0.1% vol/vol gelatine coated tissue plates in Dulbecco's modified Eagle medium (DMEM; Invitrogen, Carlsbad, CA, USA) supplemented with 20% FBS (PAA Laboratories Cölbe Germany), 2 mM glutamine, 1 mM MEM nonessential amino acids solution, 1 mM β-mercaptoethanol, leukaemia Inhibitory Factor (LIF) 1000 U/ml and an antibiotic mixture containing 100 U/ml penicillin and 100 μg/ml Streptomycin (all from Sigma-Aldrich).

Constructs 1 k-, 2 k- y 5 k-*mTert*-GFP were transfected by electroporation in mouse stem cell lines R1 and MAR1 as follow: ten micrograms of the linealized mTert-EGFP construct were electroporated into 3 × 10^6 ^cells using a Multiporator (Eppendorf, Hamburg, Germany) and a pulse of 300 V, 500 μs. Cells were allowed to recover for 24 h before G418 was added to a final concentration of 150 μg /ml. Cellular clones were selected by geneticine resistance over 7 days. At least ten clones of every construct were taken up for further analysis. Clones were disaggregated by trypsinization and allowed to attach on 96 well cell culture cluster, maintained for 2 days in LIF (Leukemia Inhibitory factor)-supplemented media until colonies were formed. Then LIF was removed from the culture media to allow the differentiation into embryoid bodies (EBs) and different cellular types.

For the promoter assays, transient transfections of the three transgenic constructs were performed into R1 ES cells by electroporation as indicated above, and the GFP activity of each construct was determined by flow cytometry using FACSCalibur System and CellQuest software. Cells transformed with an EGFP plasmid without promoter were used as controls for each transfection. Three independent experiments, each performed in duplicates, were realized. The GFP mean fluorescence intensity was expressed as relative intensity per positive cell with respect to that of the promoterless construct, pEGFP.

### Generation and screening of transgenic mice

Transgenic mice (C57BL6xCBAF1) generation and identification was carried out as previously described [19]. Briefly, transgene constructs (1 k-, 2 k, and 5 k-*mTert-*EGFP) were released from the vector by restriction endonuclease digestion and purified for embryo microinjection. Transgenic founders were backcrossed to C57BL6xCBA mice to obtain homozygotic lines. PCR-positive founder animals were confirmed by dot blot analysis and both the integrity and the transgene transmission were analysed during two generations. All the animals were maintained in an animal facility using procedures and protocols that are approved by our Institutional Animal Care and Use Committee.

### Analysis of GFP mRNA expression in different tissues of transgenic mice by RT-PCR

Poly (A^+^) RNA was extracted from liver, spleen, kidney, heart, testis, ovary, and brain of transgenic mice at different ages using the Ultraspect™ RNA Isolation System following the manufacturer's instructions (Biotecx Lab. Inc., Houston, Texas, USA). The precipitated mRNA was dissolved in DEPC-treated water and then treated with 1 U of RQ DNAse I (Promega) at 37°C for 20 min to ensure that the only source of DNA in the polymerase chain reaction (PCR) was cDNA from cellular RNA. Finally, the RNA was extracted with phenol purification and ethanol precipitation, reconstituted in 50 μl of DEPC-treated water and stored at -70°C until the RT-PCR.

The RT reaction was carried out following the manufacturer's instructions of Epicentre (Tech. Corp., Madison, Wisconsin). Five micrograms of poly(A+)RNA were dissolved in water, heat-denatured (65°C, 2 min) and reverse-transcribed at 37°C for 60 min in a final volume of 25 μl containing 0.5 mM each dNTP, 0.2 μM oligo (dT), MMLV-RT (0.5 μl), RNAsin (0.2 μl), 1× MMLV-RT buffer and 8 mM DTT. After reverse transcription, PCR was performed adding 5 μl aliquot of each sample to the PCR mix containing the specific forward primers GFP-1 (5'-TGA ACC GCA TCG AGC TGA AGG G-3') and GFP-2 (5'-TCC AGC AGG ACC ATG TGA TCG C-3'), which specifically amplified a 340 bp GFP DNA. For mice Tert amplification, primers and length of the PCR product were mTert-F1 (5'-ACT TCA ACC GCA AGA CCG ACA GG-3') and mTert-R2 (5'-GGG TGG CCA TCA GTC CAG GAT GG-3') (451 bp). Amplification was carried out in a total volume of 25 μl (1× of PCR mix containing 1 u Taq polymerase, 2.5 μl 10× buffer, both from Promega, 100 μM each dNTP, 0.1 μM each primer and 2.5 mM MgCl_2_) Samples were loaded directly from ice into the heating block at 92°C to minimize the time required to reach denaturation temperatures. For semi-quantification of the mRNA, the amount of cDNA in all of the samples was equalized. In order to determine the lowest PCR cycle number that gave a reliable detectable product, a linear range of amplification was previously set. Amplification was done with an initial step of 92°C (2 min), followed by 23 cycles for β-actine and GFP, and 26 for m*Tert *of 92°C (30 sec), 59°C (30 sec) and 72°C (30 sec). The final cycle extension was at 72°C (10 min). PCR products were resolved in 1.5% TBE agarose gels, followed by staining with ethidium bromide, and visualized using UV light. The relative abundance of each PCR product was determined by quantitative analysis of digital photographs of gels using the plug-in of Image Processing Tool Kit 5.0 (Reindeer Games Inc., Asheville, NC, USA). Negative control experiments in the absence of template RNA were performed. Generation of the expected fragments was strictly dependent on the presence of RNA in the RT reaction, as demonstrated with β-actine amplification, as a non-competitive internal standard control of the RT-PCR. To minimize variability, duplicate runs were performed for each mRNA amplified and the data were averaged.

### Immunohistochemistry

Ten- to twenty-micrometer cryosections were blocked with 5% goat serum in TBST (Tris 0.1 M, NaCl 150 mM, pH 7.5, Triton 0.1% vol/vol blocking solution), then incubated for 1 hour at room temperature (r.t.) with anti-GFP rabbit polyclonal antibodies (1:600. Molecular P robes, Eugene, OR. USA) diluted in TBST 5% goat serum. Sections then were washed three times with TBS (Tris 0.1 M, NaCl 150 mM, pH 7.5) and incubated with highly cross-adsorbed Alexa 488 anti-rabbit secondary antibodies (1:500. Molecular Probes) for 1 hour at r.t. Finally, sections were counterstained with Hoechst 33342 (Molecular Probes), and mounted with Aqua Polymount mounting media (Poly-Labo).

### Analysis of transgene expression in pluripotent stem cell isolated from testis of transgenic mice

ES-like colonies and germinal stem cells were obtained from neonatal and adult testis cells which were collected from a newborn transgenic 1 k-*mTert-*GFP mouse line of C57BL6/CBA F1 background (0–3 days old) and from 21–28 days old mice. Testis cell culture was performed according to the previously published protocol [20] with slight modifications. Briefly, testis cells were digested with 1 mg/ml collagenase (type IV, Sigma-Aldrich corporation St. Louis, MO, USA) for 10 min at 37°C, followed by 0.05% vol/vol trypsin/0,1 mM EDTA digestion for 3 min at 37°C. Testis cells were allocated to a tissue culture plate. 2.5 hours later, floating cells were recovered and passed to secondary culture plates on mitomycin-C treated (Sigma-Aldrich) mouse embryonic fibroblast (MEF) cell in 0.1% vol/vol gelatine coated tissue plates in Dulbecco's modified Eagle medium (DMEM; Invitrogen, Carlsbad, CA, USA) supplemented with 20% FBS (PAA Laboratories Cölbe Germany), 2 mM glutamine, 1 mM MEM nonessential amino acids solution, 1 mM β-mercaptoethanol, leukaemia Inhibitory Factor (LIF) 1000 U/ml and an antibiotic mixture containing 100 U/ml penicillin and 100 μg/ml Streptomycin 20 ng/ml mouse epidermal growth factor, 10 ng/ml human basic fibroblast growth factor, 10 ng/ml recombinant rat glial cell line-derived neurotrophic factor (GDNF) (all from Sigma-Aldrich).

### Analysis of transgene expression in neuroesferes obtained from E14 foetus brain of transgenic mice

Fore brain of E14 foetus of 1 k-, 2 k, and 5 k-*mTert*-EGFP transgenic mice were dissected and cut in small pieces. The tissue was suspended in HBSS (Sigma) with DNAsa 0.005% wt/vol (Sigma) and Papain 20 U/ml (Roche). Digestion was carried out at 37°C with gentle agitation every five minutes during 15 minutes. Digested tissue was centrifuged and pellet was suspended in DNAsa HBSS and mechanically dissociated with decreasing fire polished Pasteur pipettes. Cells were centrifuged twice with DNAsa HBSS and pellet was suspended in DMDM:F12, N2 supplements, AANE, Hepes 10 mM, Glucose 6 g/l, Albumax 0.1% vol/vol counted and seed at 400.000 cells/ml in the same medium plus 20 ng/ml of FGF (AbCys) and 20 ng/ml of EGF (AbCys). Neurospheres were mechanically triturated and diluted 1/5 weekly.

## Results

### Analysis of nucleotide sequence of human and mouse *TERT *promoter

To investigate the relationship between mouse and human *TERT *promoter, homology comparison was carried out between 4489 pb of the *mTert *(AF121949) and 3996 bp of *hTERT *(AF097365) sequences (Fig. 1). We have analyzed the presence of the transcription factor binding sites (SP1, c-Myc, Zap3 and MZF2), and the presence of CpG motif (Fig. 1). No significant sequence identity could be identified except for the 55 pb immediately upstream of the ATG, and another 500 bp located from position -1.4 in mouse and -1.6 in human sequences (Fig. 1). The human and mice TERT sequences have a core promoter of 220 bp with three putative SP1 and two c-Myc. The mouse promoter has another 2 c-Myc in the first -1 kb upstream of the ATG and does not have any more SP1, meanwhile the human promoter presents 7 SP1 located along the promoter. Another difference is that the human promoter has 4 inhibitors MZF2 located between -0.5 and -0.8 kb, and mouse promoter has three MZF2 located between -1.8 and -2.5 kb. Moreover, only the Zap3 binding site present in the core promoter closer to the initiating AUG codon, is conserved between human and mouse promoters.

The analysis of CpG islands showed a different pattern between *hTERT *and *mTert *promoters. The human promoter has three CpG islands covering in total more that 1.6 kb in length meanwhile in the mouse promoter a single CpG island covers 0.4 kb in length (Fig. 1).

### ES cell differentiation modifies *mTert *expression

To identify relevant elements for the regulation of *mTERT *we designed three constructs (detailed in the Fig. 1) with different promoter regions. The 1 k-mTert-GFP contains the proximal region of the promoter where all the transcription activator elements of the promoter reside (2 regions of c-Myc binding, 2 regions of Zap3 binding, and 3 regions recognized by proteins from the sp1 family). This promoter also contains the single CpG island of the 4.5 kb promoter (Fig. 1). Construct 2 k, includes, in addition to this proximal region, two of the three regions of MZF2 (myeloid zinc finger protein) binding, that reduces the transcriptional activity of the promoter. Construct 5 k contains 4.5 Kb of the mouse promoter region.

To analyze the promoter activity of the 5' flanking *mTert *region, the three transgenic constructs were transiently transfected into R1 ES cells, and the GFP activity of each construct was determined by flow cytometry. More than twofold significant increase in GFP activity was found in cells transfected with the three *mTert *promoter constructs. The shorter and the longer constructs showed better transcriptional activity compared to the 2-kb promoter (Fig 2).

**Figure 3:**
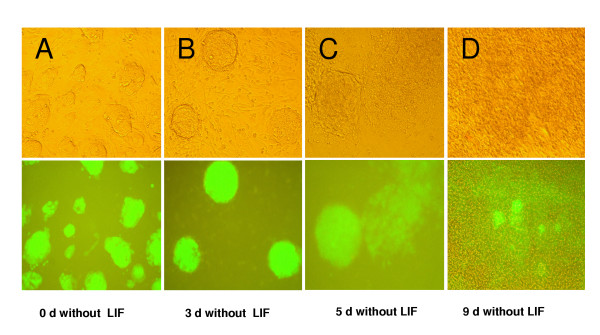
Sequence of microphotograph of ES cells transformed with 5 k-*mTert*-EGFP during differentiation induced by LIF removal. (A) ES cell expressing EGFP; (B) three days after LIF removal the ES cells form embryonic bodies (EBs); (C) Differentiation of EBs showing a diminishes on fluorescence intensity; and (D) next stage of differentiation showing a loss of fluorescence; in some wells groups of EGFP expressing cells remain immersed into differentiated colonies.

**Figure 4:**
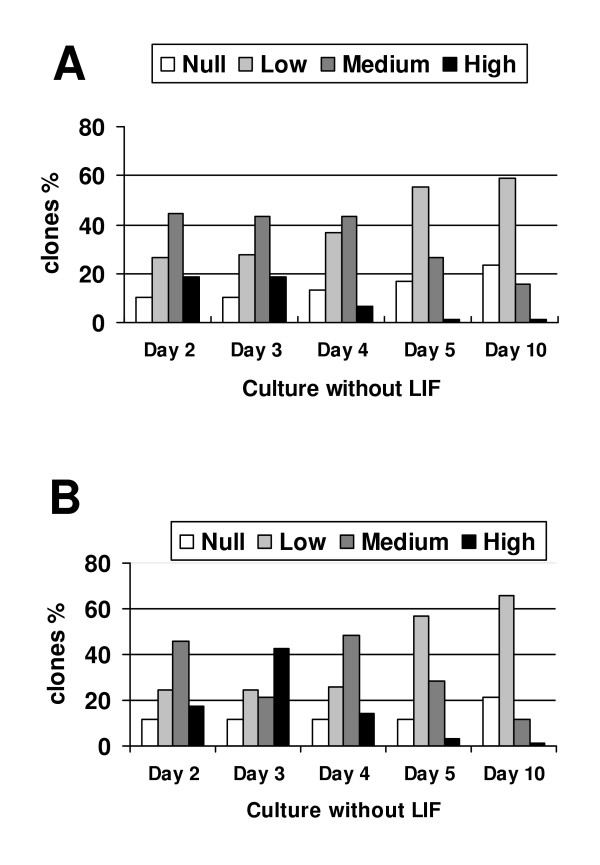
Levels of GFP expression (none, low, medium or high) in the different 5 k-*mTert*-GFP cellular clones derived from B6D2 and R1 ES cell lines. (A) 5 k*mTert*-GFP expression in 76 B6D2 clones, and (B) 5 k*mTert*-GFP expression in 85 R1 clones. GFP expression increased during 2–3 d after removal of LIF and subsequently diminished in parallel to EBs differentiation.

To analyze the regulation of *mTert*-EGFP during ES cell differentiation, we produced *mTert*-EGFP marked cell lines by stably transfecting these three constructs into murine ES cells (R1 and MAR1). The ES cell in presence of LIF expressed EGFP but only 3 days after removal of LIF, the ES cells were transformed into embryoid bodies (EBs) (Fig 3). During this process EGFP expression increased (Fig 4). In the next step of the differentiation process, where EBs evolved into differentiated cells, EGFP expression was lost. However, in some wells, groups of EGFP expressing cells remained immersed into differentiated colonies, even 9 days after LIF removal (Fig. 3). No differences were observed between the 3 constructs.

To analyze the possible differences between ES cell lines, we transformed 5 k-*mTert*-GFP in two ES cell lines (R1 and MAR1). Similar GFP expression levels were observed in the different 5 k-*mTert*-GFP cellular clones derived from B6D2 (76 clones) or R1 (85 clones) ES cell lines (Fig. 4).

### Establishment of transgenic mice lines and expression of EGFP in transgenic tissues

**Figure 5:**
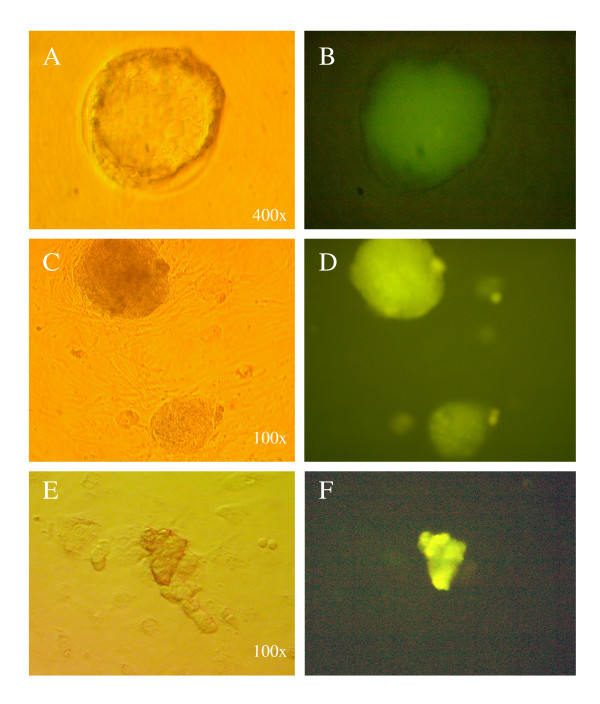
Expression of 1 k-*mTert*-EGFP transgene at blastocyst stage (A, B) and ES-like colonies (C, D) and germinal stem cells (E, F) generated from neonatal testis cells.

Eight lines of transgenic mice carrying the 1 k-*mTert*-EGFP transgene, 3 carrying the 2 k-*mTert*-EGFP transgene and 5 carrying the 5 k-*mTert*-EGFP transgene were obtained. We first analyzed the EGFP expression at the blastocyst stage under fluorescent microscopy, finding similar levels of expression in all the lines (Fig 5A–B). Then, we analyzed EGFP expression during foetal development using inmunocytochemistry from E8 to E19 dpc embryos. Only some lines showed a weak fluorescence in E13 dpc embryos in the germinal ring. No expression was detected in other tissues. For the rest of the experiments, two lines of each construct were selected based on two criteria, first that the transgene were integrated into an autosomal chromosome and could be transmitted stably with a transgene transmission ratio of 50%, and second that EGFP expression at the blastocyst stage and in E13 germinal ring were detectable (Table 1). We then examined EGFP mRNA expression in liver, spleen, kidney, heart, testis, ovary and brain of the 6 transgenic lines selected (Table 1). The mRNA expression of the three construct was lower than the endogenous *mTert*, but it recapitulated the endogenous *mTert *transcription pattern.

**Table 1:** *mTert*-EGFP transgene expression in different tissues

	1 K *mTert*-EGFP		2 K *mTert*-EGFP		5 K *mTert*-EGFP		Mouse *Tert*
Tissue	4	15	3	5	7	50	
Liver	++	+	+	-	+	+	+++
Spleen	++	+	+	+	+	+	+++
Kidney	+	-	-	+	-	+	+
Heart	+	-	-	-	+	-	+
Testis	++	++	++	+	+	++	++++
Ovary	++	+	++	+	+	+	++
Brain	+	-	+	-	+	-	+

-, No expression; +, relatively low level of expression; ++. Moderate expression; +++, high level of expression.

We then analyzed the fluorescent emission of endogenous EGFP under fluorescent microscopy and expression of EGFP protein using specific antibodies (see Material and Methods) of different tissues (bone marrow, liver, spleen, kidney, heart, testis, ovary and brain) of neonates and adult mice. No endogenous fluorescent expression of GFP was detected in any tissue. Expression of EGFP protein was also undetectable during foetal development with anti-GFP antibodies, corroborating the low levels of RNA found by RT-PCR studies.

### Expression of EGFP in cultured cells generated from transgenic embryos or tissues

We then analyzed if our three constructs could express detectable amounts of GFP (to be detectable under conventional fluorescent microscopy) in populations of stem cells generated and expanded *in vitro *from embryonic, foetal, or adult transgenic tissues. The fluorescent expression of EGFP driven by the three constructs was visualized at the blastocyst stage (Fig. 5A–B) and new ES cells lines generated from them (Fig. 5C–D); also it was visualized in ES-like colonies and germinal stem cells generated from neonatal and adult testis cells (Fig. 5 E–F).

**Figure 6:**
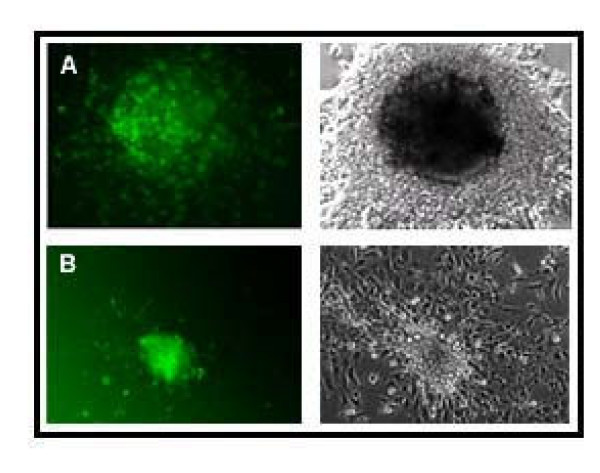
Fluorescence and contrast phase images of 10DIV cultures of forebrain foetus from 1 k-*mTert*-EGFP (A) and 5 k-*mTert*-EGFP (B) transgenic mice.

Fluorescence was detectable in cultures generated from E14 foetus brain cells derived from 1 k-mTert-EGFP (Fig 6A) and 5 k-*mTert*-EGFP mice (Fig 6B). In both cases it could be visualized at least until 10DIV (Fig 6). FACs analysis at 48 h after seeding showed a more intensive fluorescence in 5 k-*mTert*-EGFP cells compared to those of 1 k-mTert-EGFP origin (data not shown).

## Discussion

While there is much information about the human *TERT *promoter, little work has been carried out with *mTert *promoter [2,21,22]. Our results indicate that there are considerable differences between *mTert *and *hTERT *promoters, and that, at least in ES cells, the regulation of the transcriptional activity of the *mTert *promoter, in contrast with its human homologue, resides primarily in the proximal region. The differences between the two species may in fact aid our understanding of the function for telomerase regulation.

The differences in CpG islands may indicate a higher regulation of *hTERT *by methylation. It has been demonstrated that the epigenetic regulation of *hTERT *during differentiation is meditated by histone acetylation and cytosines methylation in the *TERT *promoter. The differences between human and mice *Tert *promoters may indicate the differences in regulation of these genes between both species. This could explain in part the differences in senescence and immortalisation between human and mouse primary culture cells. If the regulation of *mTert *expression represents a key gatekeeper for the transition to an immortal phenotype in the mouse, as it appears to be for human cells, then the continued expression of *Tert *in mouse cells and tissues could be an important permissive factor enabling their cancer-prone phenotype. It has been proposed that telomerase length is differently regulated in both species [23]. Another difference is the presence of Sp1 binding sites. *mTert *only has 2 sites very proximal to the ATG, whereas *hTERT *has at least 9 Sp1 binding sites dispersed along the 4 kb promoter. The clustering of Sp1 sites is a common event in promoters of TATA-less genes [24]. It is becoming increasingly clear that full transcriptional activity of the *hTERT *promoter requires the SP1 transcription factor [25,26]. There are also differences between Ap-1 sites. Recently it has been demonstrated that the species-specific function of Ap-1 in *TERT *expression may explain in part, the difference in transcriptional suppression of telomerase activity between normal human and mouse somatic cells [27].

One common thing between both promoters is the presence of c-Myc binding sites. It has been previously reported the presence of c-Myc and Sp1 binding sites in *hTERT *[28], and it has been also shown that c-Myc activates telomerase, an effect attributed to direct interaction of c-Myc with the *hTERT *promoter [29]. *mTert *promoter contains only some conserved regions compared to *hTERT *promoter, in particular the c-Myc binding region [21], suggesting that activation of telomerase by c-Myc could be a general mechanism conserved among mice and man. Only one Zap3 binding site is conserved between human and mouse promoters, suggesting that this site may be potentially significant in the regulation of *Tert *promoter activity. Recently it has been suggested that Zap3 binding sites play a role in controlling the absolute levels of *mTert *transcription [30]. The other common binding motif identified is the MZF (myeloid zinc finger protein 2). Deletion analysis of *hTERT *has shown that these motifs are in a negative regulatory region [31]. In addition, the over-expression of MZF2 in 2 human cell lines led to the down-regulation of transcriptional activities of *hTERT *promoter, indicating that MZF binding sites may act as negative regulatory elements and regulate the expression of *hTERT *[31]. In humans, this region has been named as intermediate promoter region and it has been determined that it strongly reduced *hTERT *promoter activity [32]. *mTert *promoter has three binding sites for MZF2, we have found also an effect of the presence of the region containing these elements on the expression of our construct, indicating a similar function of this repressor binding sites between species. Our results agree with Armstrong et al. [30] and confirm that the principal transcriptional regulation sites are present in the 1 k-*mTert *promoter.

The regulation of the transcriptional activity in ES cells of the *mTert *promoter, in contrast with its human homolog, resides primarily in the proximal region to the coding region. The sequence divergence between the human and mouse telomerase RNA gene promoter regions may explain the differences in telomerase regulation between human and mice species. Because the final function of the telomerase in both species is the same, the differences between the two promoters may in fact aid our understanding of the function for telomerase regulation.

During the process of differentiation no differences were observed between the 3 constructs, indicating that the 1 k promoter contains all the necessary elements for the correct regulation of *mTert *expression in ES cells. The increase in the expression of EGFP we observed during the formation of EBs could be a consequence of the enhanced cell proliferation at this period. After EBs stage, the expression of EGFP decreases during the process of differentiation, suggesting that there is a close correlation between the down-regulation of *mTert *and the differentiation process. Similar results have been reported by [2] during haematopoietic differentiation of ES cells. Using a construct with a similar 4.5 kb *mTert*, they found that those cells expressing high levels of transgenic construct also showed a significant telomerase activity. We have found here that only the first 1 kb of the promoter is necessary to induce the same phenotype. In vivo experiments confirmed this similar pattern, equivalent expression levels were found among transgenic lines produced with the 1 K, 2 K or 5 K promoter.

In our transgenic model no fluorescent expression of the *mTert*-EGFP construct could be identified in adult tissues. This suggests either a scarce presence of adult stem cells in the total population of adult tissues, with undetectable expression levels in physiological conditions, or a tight repression system of this promoter. A similar effect was observed [2,17] using h*TERT *promoter. However, when tumours were induced in their transgenic models, expression levels became detectable. In our case we were able to recover expression levels when new stem cell lines were established from embryonic, foetal, newborn and adult tissues obtained from transgenic animals of all the lines selected. This suggests that the construct was functional and that its activity in adult tissues migth not be a consequence of transgene inactivation but a consequence of the mechanism of telomerase physiological repression in the adult tissue. *mTert *activity is recovered when repression mechanisms disappear as a consequence of *in vitro *culture conditions that stimulates cell proliferation and undifferenciation like those used for stem cell generation. This up-regulation of *Tert *activity observed in our new stem cell originated under *in vitro *culture conditions and the up-regulation observed in tumours are indicating that *Tert *expression is related with a transformation stage of the cells that confers a survival advantage.

## Conclusion

*mTert*-EGFP system is an excellent marker for the identification of the promoter elements that regulate *mTert *activity during stem cell differentiation

The 1 kb promoter upstream of the initiating ATG codon contains all the regulatory elements to regulate telomerase expression in ES cells during *in vitro *loss of pluripotencia. The regulation of the transcriptional activity in ES cells of the *mTert *promoter, in contrast with its human homolog, resides primarily in this proximal region of the coding region. The sequence divergence between the human and mouse telomerase RNA gene promoter regions may explain the differences in telomerase regulation between human and mice species

This transgenic mouse model generated is a potential reporter system for the selection and isolation of stem cells generated from embryos (ES cells), from foetus (neuroesferes) and from newborn tissues (ES-like and germinal stem cells).

## Authors' contributions

MAR and EP performed most the experiments and prepared the manuscript. AVD, ARG and JMT performed the experiments in neuroespheres and critically reviewed the manuscript. MN performed the inmunocytochemistry analysis of EGFP expression during foetal development and critically reviewed the manuscript. BP and AGA supervised all the work and assisted in writing the manuscript.
